# Funding and remuneration of interdisciplinary primary care teams in Canada: a conceptual framework and application

**DOI:** 10.1186/s12913-017-2290-4

**Published:** 2017-05-15

**Authors:** W. Dominika Wranik, Susan M. Haydt, Alan Katz, Adrian R. Levy, Maryna Korchagina, Jeanette M. Edwards, Ian Bower

**Affiliations:** 1 School of Public Administration, Dalhousie University, Halifax, Canada; 2 Faculty of Management, Dalhousie University, Halifax, Canada; 3 Department of Community Health Sciences, Department of Family Medicine, University of Manitoba, Winnipeg, Canada; 4 Department of Community Health and Epidemiology, Dalhousie University, Halifax, Canada; 5 Provider Compensation and Strategic Partnership Branch, Alberta Health, Edmonton, Canada; 6 Primary Health Care and Chronic Disease, Winnipeg Regional Health Authority, Winnipeg, Canada; 7 Primary Care, Nova Scotia Department of Health and Wellness, Halifax, Canada

## Abstract

**Background:**

Reliance on interdisciplinary teams in the delivery of primary care is on the rise. Funding bodies strive to design financial environments that support collaboration between providers. At present, the design of financial arrangements has been fragmented and not based on evidence. The root of the problem is a lack of systematic evidence demonstrating the superiority of any particular financial arrangement, or a solid understanding of options. In this study we develop a framework for the conceptualization and analysis of financial arrangements in interdisciplinary primary care teams.

**Methods:**

We use qualitative data from three sources: (i) interviews with 19 primary care decision makers representing 215 clinics in three Canadian provinces, (ii) a research roundtable with 14 primary care decision makers and/or researchers, and (iii) policy documents. Transcripts from interviews and the roundtable were coded thematically and a framework synthesis approach was applied.

**Results:**

Our conceptual framework differentiates between team level funding and provider level remuneration, and characterizes the interplay and consonance between them. Particularly the notions of hierarchy, segregation, and dependence of provider incomes, and the link between funding and team activities are introduced as new clarifying concepts, and their implications explored. The framework is applied to the analysis of collaboration incentives, which appear strongest when provider incomes are interdependent, funding is linked to the team as a whole, and accountability does not have multiple lines. Emergent implementation issues discussed by respondents include: (i) centrality of budget negotiations; (ii) approaches to patient rostering; (iii) unclear funding sources for space and equipment; and (iv) challenges with community engagement. The creation of patient rosters is perceived as a surprisingly contentious issue, and the challenges of funding for space and equipment remain unresolved.

**Conclusions:**

The development and application of a conceptual framework is an important step to the systematic study of the best performing financial models in the context of interdisciplinary primary care. The identification of optimal financial arrangements must be contextualized in terms of feasibility and the implementation environment. In general, financial hierarchy, both overt and covert, is considered a barrier to collaboration.

**Electronic supplementary material:**

The online version of this article (doi:10.1186/s12913-017-2290-4) contains supplementary material, which is available to authorized users.

## Background

Mature health systems are placing increased emphasis on Interdisciplinary Primary Care Teams (IDPC Teams) to deliver primary care [1]. This approach has been described as more appropriate in addressing the health needs of populations by creating more comprehensive care options and increasing continuity and coordination [2–6]. The financial structure of IDPC teams has been recognized as an important contributor to team functioning and therefore a key concern to policy makers in this area.

The Canadian health care system is publicly funded; close to 70% of all health expenditures are tax financed [7]. The system is governed by a national Canada Health Act, which stipulates the broad conditions that Provinces/ Territories must comply with in order to qualify for federal budget transfers. The specifics of health care budget allocation are resolved at the level of the Provinces/Territories. In many cases, especially at the primary care level, delivery of services is by private providers. This results in a set of 13 similar, yet unique health systems in Canada, making it an interesting case study from the international perspective.

Primary care physicians are gatekeepers to the health care system; patients typically cannot access specialist services without a prior primary care consultation. Primary care physician services are covered by the public insurance system, meaning patients do not face out of pocket payments for services that are defined as “medically necessary”. The Canada Health Act requires coverage for services delivered by physicians, but coverage of services provided by other primary care providers is optional. Therefore there is no mechanism to ensure a unified national approach to the funding of IDPC teams or remuneration of providers within these teams.

Approximately half of all physicians are generalists/ family physicians [8]. On average, family physicians report billing 32.3% of their incomes from fee-for-service (FFS), 6.3% from salaries, 4.9% from sessional rates, 45.9% from blended models, and 10.6% from other remuneration methods [9].

Primary care reform in Canada has been ongoing over the past 16 years, with a large system-wide impetus in 2000 and 2006, when the Federal government provided support to Provinces/ Territories to redesign the delivery of primary care through the Primary Care Transition Fund [7]. This was operationalized differently across regions in how IDPC teams were designed and implemented [10, 11]. Some provinces, such as Alberta and Ontario, introduced system wide policy frameworks to which primary care providers were invited to adhere [12]—a top-down approach. Other provinces, such as Nova Scotia, introduced policies in response to existing changes in front-line delivery—a bottom-up approach. The resultant financial models are the focus of this study.

Using primary qualitative interview data, as well as secondary online data-sources from three Canadian Provinces we develop a conceptual framework of funding and remuneration models in the IDPC team setting. We apply the framework to the analysis of incentives created, expected impacts on collaboration, and the assessment of optimal financial models in a variety of contexts. Finally, we discuss issues associated with the implementation of optimal models on the basis of qualitative data from a research roundtable discussion.

### Literature

Literature focused on IDPC teams typically omits discussion of funding and remuneration methods, except to mention their importance. For more than four decades, the emphasis has been on how team-based care can offer solutions to system problems such as a growing patient population and shortages of trained personnel [13]. It has been argued that well-functioning teams, when compared to typical sole profession practices, appear to have a number of advantages. IDPC Teams can create conditions for improved health outcomes, improved clinical performance, higher quality of care, and improved chronic disease management [2–6, 14–20]. IDPC teams may be preferred by patients [3–5, 21], and by providers [3, 4, 6, 18, 22, 23]. Lastly, some suggest improvements in system level outcomes, such as efficiency of resource use and reduced fragmentation [3, 5, 6, 23, 24].

Studies have also focused on identifying a number of organizational factors that influence the functioning of IDPC teams. Facilitators of team functioning described in the literature include supportive, clear and transparent processes, institutional reinforcements, and a more elusive sense of togetherness [4, 6, 15, 18, 22, 23, 25–31]. Barriers to team functioning described in the literature include insufficient education and training, the mismanagement of resources and team diversity (e.g. creation of silos or imbalanced power relationships or pay status between providers) and miscommunication [15, 26–28, 30, 32–37].

However, the literature is mostly silent on the description or effects of financing models for IDPC teams, aside from indicating that these are important [38–40]. The design of funding and remuneration models remains understudied in terms of options and impact on team functioning. Financing is considered important, with very little analysis or discussion of optimal methods [41].

There is a lack of descriptive, comparative or evaluative studies of various approaches to the funding of teams and remuneration of providers within the primary care context. Several studies focus on the health care outcomes under the different care models with varying remuneration methods for physicians in Canada [42–46]. One draws a distinction between practice and provider level financial incentives, but not the interaction between them [47]. One conceptual framework provides a broad overview of incentives created by various remuneration and funding mechanisms at the provider or the organization level, and their ability to support a variety of policy goals, but does not discuss the interactions between various levels of incentives [24]. While a large strand of literature has focused on the remuneration of individual providers (examples of systematic reviews include [48–51]), this is generally not addressed at the team level. Some observe that the variety of remuneration methods within teams stand in the way of effective team practice [52]. Few studies incorporate a discussion about the remuneration of non-physician providers [1, 53]. In the majority of literature the issues of interplay between team funding and provider remuneration, and between remuneration of physician and non-physician providers are ignored. Our study partially fills these gaps by providing a conceptual framework that facilitates analysis of approaches to team financing; the framework is applied to approaches used in three Canadian provinces, with focus on incentives for collaboration between providers.

## Methods

### Study design

This is a qualitative study using semi-structured interviews, a research roundtable discussion, and a document review. Data collection was conducted between January 2014 and October 2014. The study protocol was approved by the research ethics committees at nine district health authorities in Nova Scotia, at the University of Manitoba, and at Alberta Health. The study protocol and reporting followed the COREQ guidelines (see Additional file 1).

The study was conducted using an integrated knowledge translation approach, which relies on the continuous involvement of stakeholders is all stages of the research process. The research team included policy decision makers from each of the three Provinces, who shaped the research questions, facilitated data collection, provided data, and participated in analysis and interpretation.

### Sampling

We relied on purposive sampling to recruit leaders overseeing IDPC Teams, such as executive directors, directors, and/or managers (titles varied by Province). In Nova Scotia, respondents were at the health authority level and oversaw more than one clinic. In Alberta, we interviewed executive directors of Primary Care Networks of varying sizes, most with multiple clinics, including one or more sites. In Manitoba, we interviewed primarily managers of individual clinics. Potential respondents were identified by the Department of Health and Wellness in Nova Scotia, by the Ministry of Health in Manitoba, and by an online search for contacts of Primary Care Networks in Alberta. For the Research Roundtable, we invited policy decision-makers from the three Provinces, as well as one to two interview respondents from each province.

### Data collection and analysis

The descriptive component of our study was developed using online policy documents across Canada (see Appendix), academic studies and qualitative interviews. The evaluative component relied on the qualitative interviews, as well as a research roundtable. Specifically, the document review aimed at describing the types of IDPC Teams across Canada, the interviews aimed at the assessment of merits and demerits of various options, and the research roundtable aimed at the discussion of implementation issues. Given the qualitative nature of the study, interview respondents and roundtable participants were able to address all issues (description, assessment, and implementation) at their discretion.

Semi-structured qualitative interviews (Additional file 2) were held between January and May 2014 with 19 respondents, who represented six PCNs in Alberta, eight clinics in Manitoba, and five district health authorities in Nova Scotia (four at the regional level, and one discussing four individual clinics separately). Qualitative data related to the description of the structure of teams were analyzed using a framework synthesis approach [54], which was structured around the a priori framework that had also guided data collection (Additional file 3). Qualitative data related to the assessment of strengths and weaknesses and implementation issues were analysed using a grounded theory approach [55, 56], with focus on emergent themes not already covered by the a priori framework.

Qualitative components of interview responses were organized into a matrix. Responses within individual matrix elements were coded and synthesized (goals, logistics, organization, etc.). The factual information within the matrix was supplemented with/corroborated by data from the document reviews. In addition, respondents completed a table outlining the composition of their team and the method of payment offered to types of providers. Data identifying team composition, remuneration method, funding source were compiled into a table to support the framework synthesis approach (Additional file 3).

The research roundtable took place on October 27/28, 2014 at Dalhousie University in Halifax, Nova Scotia (Additional file 4). The roundtable had 14 participants, of which four are co-authors of the paper, five had previously participated as an interview respondent, and five were new to the study. Participants discussed a draft framework, extant financial models in the three provinces, their strengths and weaknesses with emphasis on creating collaboration incentives, and implementation issues as perceived by policy makers versus managers of teams. The roundtable was transcribed and coded. Thematic analysis was used to analyse the transcriptions.

Iteratively, to guide us during data collection and as a result of data analysis, an analytical and conceptual framework was developed [57, 58]. The framework consists of two elements, a spectrum of funding models, and a typology of nine distinct types of team funding. Both are described in detail in the results section. The purpose of the framework was to analyze the qualitative data in this study, conceptualize funding approaches in a more general sense, and support the collection and analysis of further empirical data.

## Results and discussion

### Characteristics of teams described by respondents

Profiles of teams described by interview respondents are summarized in Table 1. Participating Primary Care Networks in Alberta ranged from one to 86 clinics, and between 2 and 385 physicians, 3 to 55 nurses, and 1 to 39 other providers. In each case, the PCN receives funding through Alberta Health to pay non-physician and non-clinical staff. The funding amount is based on the patient numbers of individual physicians. Physicians bill on a FFS basis from a separate funding channel. Respondents in Manitoba each described one clinic/ team with a membership of 1 to 28 physicians, 1 to 9 nurses, and 1 to 43 other providers. Physicians in Manitoba bill FFS from the Department of Health, or receive salaries from the Health Authority, or a mix of these two. The predominant method of remuneration of other providers is salary. Respondents in Nova Scotia described between 1 and 8 clinics, consisting of 1 to 12 physicians, 2 to 10 nurses, and 3 to 6 other providers. In the clinics described here, physicians in Nova Scotia receive salaries through an alternative funding plan from the Department of Health, and other providers receive salaries from the Health Authority.

**Table 1:** Remuneration and Funding Profiles of Study Networks/ Teams

ID*	Number of clinics	Number of patients**	Physicians			Nurses/ Nurse Practitioners			Other care providers			Non-provider staff		
			Number	Remuneration	Source***	Number	Remuneration	Source***	Number	Remuneration	Source***	Number	Remuneration	Source***
AB1	86	387,000	385	FFS	DH	55	salary	PCN	39	salary	PCN		salary	PCN
AB2	37	100,000	82	FFS	DH	51	salary	PCN	13	salary	PCN		salary	PCN
AB3	12	23,000	21	FFS	DH	10	salary	PCN	2	salary	PCN		salary	PCN
AB4	19	120,000	82	hourly	DH	12	salary	PCN	13	salary	PCN		salary	PCN
AB5	1	20,000	2	FFS	DH	3	salary	PCN	1	salary	PCN		salary	PCN
AB6	29	87,000	65	FFS	DH	27	salary	PCN	9	salary	PCN		salary	PCN
MB1	1	–	4	FFS/salary	DH/HA	8	salary	HA	43	salary	DH/HA		salary	DH/HA
MB2	1	850	3	FFS/salary	DH/HA	6	salary	HA	4	salary	HA		salary	HA
MB3	1	–	3	FFS/salary	DH/HA	4	salary	HA	ns	salary	HA		salary	HA
MB4	1	–	1	FFS	DH	4	salary	HA	2	salary	HA/other		salary	HA
MB5	1	–	1	salary	HA	9	salary	HA	4	salary	HA		salary	HA
MB6	1	–	6	salary	HA	5	salary	HA	3	salary	HA		salary	HA
MB7	1	–	28	FFS/salary	DH/HA	9	salary	clinic (FFS)	2	hourly	patients	ns	ns	ns
MB8	1	30,000	19	FFS	DH	1	salary	clinic (FFS)	1	salary	HA		salary	clinic (FFS)
NS1	8	13,000	ns	salary/FFS	APP/ DH	ns	salary	HA + other	ns	salary		ns	salary	HA
NS2	1	ns	1	salary	APP (DH)	2	salary	HA	3	salary	HA	ns	salary	HA
NS3	3	11,000	~6	salary	APP (DH)	~3	salary	HA	–	–	–	ns	salary	HA
NS4	4	14,000	13	salary	APP (DH)	10	salary	HA	6	salary	HA	ns	salary	HA
NS5	7	10,000	12	salary	APP (DH)	8	salary	HA	ns	salary		ns	salary	HA

*Respondent ID indicates the province—*AB* is Alberta, *MB* is Manitoba, *NS* is Nova Scotia

**Respondents in MB indicated that a panel size was not recorded. One clinic is a specialty clinic with 850 clients. One clinic estimated the number of potential patients in the geographical catchment area

***Funding sources are coded as: *DH* Department of Health (names vary across provinces and over time); *HA* Health Authority (names vary across provinces and over time), *ARP* Alternative Payment Plan (includes any salary contract for physicians, often accompanied by shadow billing requirements); *PCN* Primary Care Network (whole team or network grant), *ns* number not specified (e.g. some, several)

### Defining the interdisciplinary primary care team

Sixteen respondents indicated that the interdisciplinary primary care team brings together different healthcare providers or health professionals who work together. Nine explicitly stated that the care is organized around the patients’ or clients’ needs, and five indicated that an IDPC team serves the same client population (patient panel or roster). In addition, respondents mentioned shared goals, communication, co-location, and comprehensive and seamless care. For the purposes of this study, we define “collaboration” to encompass the working together of different health professionals to achieve shared goals [59]. Respondents described IDPC teams as centered on client needs, and one respondent characterized the physician as the core of the team.

“*Different health professionals of different disciplines working together to better serve the client*.”

“… *number of allied health workers who are working alongside the physician…*”.

### Goals of the interdisciplinary primary care team

Goals described by interview respondents fell into three categories: (i) care for particular conditions; (ii) achievement of health system goals; and (iii) workplace improvement. For example, respondents discussed the management and self-management of chronic conditions, provision of targeted services such as obstetrics, HIV care, sexual health, improvements in access for patients or work-life balance for providers, and other goals.

### Spectrum of financial hierarchy and financial integration (conceptual framework)

**Fig. 1:**
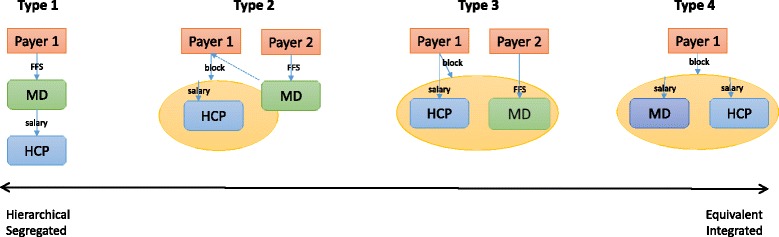
Spectrum of Financial Hierarchy and Integration

The profiles of the funding and remuneration approaches described by respondents are captured in Table 1. Based on the descriptions of the funding models by interview respondents, we propose a spectrum of the degree of *financial hierarchy* and *financial integration* within teams/ networks (Fig. 1). On the basis of interviews, we identify these as two core aspects to characterize Weberian ideal types of financial models [57, 58]. The spectrum allows for a discussion of observed models and the extent to which they approach one of the ideal types.

Financial hierarchy refers to the degree to which incomes of some providers depend on the activities of other providers, in other words the focus is on hierarchy that is created by financial arrangements. Financial integration is the extent to which remuneration sources and resultant accountability flows are streamlined. To facilitate discussion, four discrete types in the continuum are proposed, the two extreme ends of the spectrum, and two intermediate types.

The traditional model of primary practice in the Canadian FFS environment is captured in Type 1. A physician (MD) receives FFS payments from the Ministry of Health and can, at their discretion, employ additional health care providers (HCP). At the opposite of the spectrum lies the integrated non-hierarchical model with equivalent providers, labelled Type 4. One payer allocates a block grant to a primary care team entity (clinic, network, denoted with a yellow oval), who distributes the payments to individual health care providers, including the physician. Community Health Centres across Canada most closely resemble this type (http://www.cachc.ca/).

Type 2 captures a covert hierarchy: a block grant offered to the team entity is based on the activities of the physician. The physician receives FFS payments from the same or a second payer, whereas other health care providers receive salaries from the team entity. A financial hierarchy is maintained due to the dependence of non-physician provider incomes on the activities of the physician. Type 2 fits the financial arrangements in most Primary Care Networks in Alberta, and several of the responding clinics in Manitoba.

For example, PCNs in Alberta receive a capitation payment. The associated patient roster is established on the basis of patient visits to physicians who are members of the PCN. In addition, physicians continue to bill FFS. Therefore, at the core of the funding amount to the PCN is the physician-patient interaction, even though a block fund is transferred to the PCN. A similar dynamic is observed in several clinics in Manitoba, where funding to the team is tied to the physicians’ activities and/or patient roster, or funded directly by the physicians’ FFS billings. This model was criticized by many respondents as one prioritizing physician activities and creating a dependency of all incomes on the activities of physicians, which could impede team practice. For example, there is a reluctance to delegate patients to non-physician providers as this reduces the capacity for fee-for-services billing. There is also an incentive for physicians to participate in patient visits with other providers where such participation is not necessary from the clinical perspective. Physicians were also described as being in charge, even if not officially, for example:

*“… Even though the PCN will pay the salary and put in a highly skilled person into an office, there’s pushback on that because of the risk of [the physician] not being able to charge for that patient appointment …”.*

The multiplicity of funding sources adds further complications, such as multiple lines of accountability within what is thought to be one organization, and differential amounts of payment for similar tasks. As noted by one respondent:

“*I have multiple funding sources. […] I am left sometimes with the perception that some people are getting paid a lot for the care that they provide versus perhaps what the physician might provide* …” [for the same payment].

Further toward the east end of the spectrum lies Type 3, a non-hierarchical model that retains segregation of providers through multiple funding sources. Primary Care Teams in Nova Scotia are examples of this type—an equivalent but segregated model. Funding of other providers and/or the team is not related to the activities of the physician, but physicians’ and other providers’ remuneration is drawn from different sources (e.g. health ministry versus health authority). Some teams receive block transfers, and in several instances these originate with different sources (e.g. health ministry, pharmaceutical company, special programs through another ministry, etc.) with competing priorities. The lines of accountability become blurred. Respondents noted:

*“… there’s a big challenge there for physicians in understanding that they’re a part of a bigger system that sometimes they don’t have control over, and sometimes none of us have any control over.*”

*“… we don’t have direct control over how these clinicians work in our model, so that’s a challenge …*”,

*“…They work their hours, they’re paid their salary, but there’s no control over their output, as you say, how do you get that output?* “.

The reluctance to relinquish control on the part of physicians is in part justified in that decisions about care can be delayed through a team or organizational approach:

“*… physicians generally shun the formal governance world … if a decision needs to be made, they just make it and away we go,* versus *having to wade through a number of levels of decision-making and waiting for approvals …* “.

In situations where physicians continue to receive FFS remuneration, and not salaries, respondents observed barriers to delegation:

*“… physicians don’t want her [practice nurse] doing them [services] because then that’s taking money out of their pocket …*”.

The environment for collaboration is also impeded when providers have different employment benefits due to multiple funders and funding sources:

“*The only thing is that docs have a totally separate contract. So they come in at a different vacation rate […] why do the docs start with 4 weeks’ vacation and everyone else starts at 3?* …”.

The FFS model was further seen as a barrier to collaboration mediated by the lack of space:

“… *so if you’re strictly fee-for-service, and then we’re trying to put a nurse in one of your exam rooms, so now you’re down one exam room, you know, you’ve just cut your patient load for that day in half …”.*

It should be noted that remuneration was not necessarily considered as the core motivator for providers, for example respondents noted:

“*If you are looking for financial remuneration for doing the work, this is not the organization for you*.”

*“… so it’s not all driven by who pays and who is responsible for the policy of that person …*”.

### Typology of financial models (conceptual framework)

The spectrum of financial segregation/ integration is further disentangled with a typology [57, 58, 60] and its application to a discussion of potential incentive effects on collaboration. Respondents (interviews and roundtable) agreed that collaboration was an important interim outcome, even though patient outcomes would be of ultimate interest. The alignment of incentive effects has been mentioned as important [38–40], but has not been unpacked in detail.

The typology defines two dimensions of the financial model: (i) funding—the financial compensation of the team as an entity (and degree to which this is isolated from remuneration), and (ii) remuneration—the financial compensation of providers within teams (and degree to which their incomes are dependent). Each dimension has three categories. Each type is a combination of one funding category and one remuneration category. This allows for a discussion of impact of the interplay between funding and remuneration on, for example, collaboration, to which the typology is applied in the present study.

Approaches to funding are categorized by the extent to which they are linked to the activities of the team: (i) funding that is directly pegged to the activities of the team as a whole (e.g. number of services provided by the team, number of patients serviced by the team); (ii) funding that is not linked to activities (e.g. block grants based on a geographical roster or budget priorities); and (iii) funding that is directly pegged to the activities of a core provider (e.g. a FFS payment to the physician). Approaches to remuneration are categorized by the degree of dependence between provider incomes: (i) interdependence (incomes of providers depend on the activities of other providers to the same degree, e.g. providers receive a fixed share of an activity based team fund); (ii) independence (incomes of providers are not linked to each other, e.g. all receive a fixed salary); and (iii) hierarchical dependence (incomes of some providers depend on the activities of a core provider, but not vice versa, e.g. the FFS revenue of a physician is used to pay salaries of the nurses). Eight plausible model types emerge; an example of each is provided in Table 2.

**Table 2:** Typology of Financial Models*

			Remuneration to Providers (Type of dependence between provider incomes)		
			Interdependence	Independence	Hierarchical dependence
		Impact on Collaboration	Positive	Neutral	Negative
Funding to teams (funding base)	Linked to the activities of the whole team	Positive	Patient rostered to team, providers receive a fixed share.	Patients rostered to team, providers receive fixed salaries.	Patient attached to team, P4P to individual providers.
	Delinked from provider activities	Neutral	Geographical roster, providers receive fixed share.	Geographical roster, providers receive fixed salaries.	Geographical roster, P4P to individual providers.
	Linked to the activities of one provider	Negative	Not possible.	Patients rostered to physician, providers receive fixed salaries from team.	Patients rostered to physician, physician pays others.

* Cells provide examples, not an exhaustive list

The typology can serve as an analytical tool for assessing the performance of financial models along a number of criteria, and can serve as a framework to guide the collection of further empirical data. As an example, incentives for collaboration are greatest in the top left cell, which corresponds to Type 4 in the spectrum, and lowest in the bottom right cell, which corresponds to Type 1. Each provider is motivated to collaborate with others, when the reward is tied to the performance of the whole team. Furthermore, as discussed in the previous section, a financial hierarchy discourages collaboration.

The typology allows for a discussion of optimal financial arrangements in the presence of contextual constraints. For example, in a rural setting in Canada, a patient roster would have to be geographical. The typology illustrates that this incentive neutral funding category can be coupled with an interdependence in remuneration to optimize the model type. It also highlights that the interplay between funding at the team level and remuneration of providers must be taken into account.

### Emergent implementation issues

Implementation issues were discussed primarily during the roundtable, but were also addressed by respondents during qualitative interviews. They are issues identified as important to consider during the implementation of a selected funding/ remuneration model. Four core issues emerged from the thematic analysis of qualitative roundtable and interview data: (i) budget negotiations; (ii) rostering of patients; (iii) funding for space and equipment; and (iv) community engagement.

First, the negotiations of budgets include decisions regarding the mix of providers and services, as well as the budget base (services or resources based). Provincial ministry representatives noted that evidence is insufficient with respect to the optimal mix of providers or services, and decisions were often based on convention or convenience. Team managers, some of whom were also providers, thought that the distance between budget negotiations and the front-line of care provision was inversely related to the ability for teams to respond to patient/community needs. They noted a disagreement between ministries and managers, where ministries would prefer *ex ante* planning of services based on evidence, and managers/ providers would prefer flexibility and the ability to adjust services to emergent needs of the patient population, facilitated by a resources based budget.

Second, the question of patient rosters proved surprisingly contentious. Terminology varied across provinces, from roster, to panel, to patient list and patient attachment. Some saw the roster as a suitable tool for the funder to influence services by defining targeted roster sizes per physician:

“… *all the patients in our collaborative practice are rostered to a doctor and the clinic and full time physicians are expected to have a minimum panel of 1350 patients* …”.

Others perceived the roster as a negative topic:

“… *panel management within the province […] has been a contentious issue with physicians in the province …*”

“*… [we are] very familiar with the administrative difficulties of looking at panels but more importantly, we also have the fundamental belief that this should be a patient decision*” [referring to the choice of doctor].

From the perspective of the financial model, a patient roster is necessary for any component of funding or remuneration to be capitation. For example, for a team as an entity to receive block funding, the size of its patient population must be defined. This presents a challenge, when the development of patient rosters is politically not desirable, yet the hesitations are not clearly articulated. It is also a challenge, when the funding model requires that patients be rostered to physicians, which preserves a hierarchy. Furthermore, respondents observed that physicians would “drop-in” on visits with other providers to fill the requirement of the specific patient roster model.

Third, roundtable participants discussed the problem of funding for space and equipment. Approaches to this issue varied widely across participating sites, from the physician as owner of the clinic structure, to ad hoc solutions such as space sharing with a community centre, or emergency room, and having no financial support for space. For example:

“…*physicians own their clinics … we pay a very small amount of space within their clinic* …”;

“*and the building, the space, the EMR are all funded through the district, … exam tables, you name it … the docs come and they pay us […] just to sit down and work* …”

“… *clinic space is actually what used to be a community hospital and now it is partially a collaborative practice clinic and partially a […] long term care facility* …”

“*there’s the touchy point is that no, we actually don’t provide any compensation*”. [for space]

Fourth, a desire to be responsive to community needs, and/or engage communities and patients in care planning and processes was discussed. One participant noted about community engagement “*That’s the dream, that’s what I want*.” Approaches to community engagement ranged from inclusion of patients in care decisions to engaging patients in formal planning processes.

“… *engaging the patient as part of their care and seeing them as part of the team …*”

“*… what our community health boards are meant to do […] [is] ask the public that they serve what they need* ..”,

“*There is a patient in the health authority […]* [engaged] *on a policy level …*”.

Respondents noted an emerging conflict around the issue of decision-making. Increasingly, it appeared that health authorities strive to be the decision-makers, and governments are pushing for standardization of care. This was perceived as being in conflict with the concurrent general push to involve communities and patient populations in the care-planning processes. Respondents identified these as contradictory forces, and noted emerging contradictory lines of accountability to the funder, to the patients and to the community.

## Conclusions

The goal of our paper is to create a conceptual framework for the study of financial arrangements in IDPC teams. The framework consists of a typology of provider remuneration methods, and team funding models. The framework is applied to the discussion of financial incentives for collaboration that are created in three Canadian provinces, the desirability of particular financial arrangements relative to others, and the implementation issues identified by managers and decision-makers. Consequently, our results are twofold; we provide the conceptual framework, and we provide qualitative evidence of the implications of particular financial arrangements on collaboration.

Our study indicates that incentives are strongest, when provider remuneration is interdependent and combined with a team funding model that is linked to whole team activities. The conceptually optimal arrangement must align with the context to which it is applied, however, and important implementation issues need to be considered. For example, a clear patient roster is required, which may not be feasible for political or other reasons. Furthermore, we know that non-financial incentives play an important role in motivating providers. An exploration of the interplay between financial and non-financial incentives would be an interesting follow-up study.

It is clear that a financial hierarchy impedes the collaboration function of an IDPC team. Removal of the direct hierarchy between two types of providers, however, is not sufficient. Hierarchy continues to be seen as a challenge in situations where team funding is tied to physician activities. Furthermore, an additional challenge is created through a multiplicity of funding sources that results in multiple lines of accountability. In many examples, the current financial arrangements define the physician as distinct, and often more important than other providers.

A clear conceptual framework is an important step in the building of a systematic evidence base for the design of financial models in the IDPC context. It facilitates data collection for further qualitative and quantitative studies, and provides a reference point for the discussion and comparison of results.

### Additional files

Additional file 1: COREQ. Consolidated Checklist for the Reporting of Qualitative Research—Checklist. Description of data: n/a. (DOCX 16 kb)

Additional file 2: Qualitative Interview Questions. Description of data: Questions used during qualitative interviews. (DOCX 19 kb)

Additional file 3: Qualitative Analysis Tool. Description of data: The framework used to assist with analysis of qualitative interviews. (DOCX 17 kb)

Additional file 4: Roundtable Agenda. Description of data: Content of discussion during the 2 day research roundtable data collection component. (DOCX 17 kb)

## Appendix

### Policy Documents

Manitoba Health, Healthy Living and Seniors. My Health Teams; Key Elements. Accessed online: http://www.gov.mb.ca/health/primarycare/public/myhts/elements.html.

Government of Saskatchewan. 2012. Patient centered community designed team delivery. A framework for achieving a high performing primary health care system in Saskatchewan.

Patient Centred. (2012). *Primary Health Care Framework Report.* Retrieved online at: http://www.health.gov.sk.ca/phc-framework-report.

Saskatchewan Medical Association. (2014). *Primary Health Care Team Composition.* Retrieved online at: http://www.sma.sk.ca/utility/28/primary-health-care-framework.html

Ontario Ministry of Health and Long Term Care. (2009). *Family Health Teams: Advancing Family Health Care a Guide to Physcian Compensation.* Toronto: Ontario Ministry of Health and Long Term Care.

Ontario Ministry of Health and Long Term Care. (2009). *Family Health Teams: Advancing Family Health Care a Guide to Provider Compensation.* Toronto: Ontario Ministry of Health and Long Term Care.

Ontario Ministry of Health and Long Term Care. (2009). *Family Health Teams: Advancing Family Health Care Guide for Goverance.* Toronto: Ontario Ministry of Health and Long Term Care.

Ontario Ministry of Health and Long Term Care. (2009). *Guide to Governance and Accountability: Advancing Family Health Teams.* Toronto: Ontario Ministry of Health and Long Term Care.

Ontario's Community Health Centres. (2013). Addressing the great health divide. Toronto: Ontario's Community Health Centres.

Department of Health . (2010, Nov). *A New Model of Care in Nova Scotia*. Retrieved Nov 28, 2014, from http://novascotia.ca/dhw/mocins/docs/MOCINS-evaluation-highlights.pdf.

Province of Nova Scotia. (2007, Nov 05). *Health Team Nova Scotia* . Retrieved Nov 23, 2014, from Nova Scotia Health and Wellness: http://www.healthteamnovascotia.ca/healthcare/structure.html.

Health Canada. (2007, March 1). Primary Health Care Transition Fund. Retrieved Dec 8, 2014, from Health Care System: http://www.hc-sc.gc.ca/hcs-sss/prim/phctf-fassp/index-eng.php.

Alberta Health, Primary Healthcare Branch. 2014. Strategy Highlights; Primary Healthcare Strategy. Accessed online: http://www.health.alberta.ca/documents/Primary-Health-Care-Strategy-Highlights-2014.pdf.

Alberta Medical Association. 2014. Community Engagement for PCNS: A guidebook with tools for developing a community engagement plan.

Nova Scotia Department of Health and Wellness. Collaborative care teams. 2015. Accessed June 15, http://novascotia.ca/dhw/collaborative-care-teams/

## Abbreviations

FFS: Fee for Service
HCP: Health Care Provider
IDPC: Interdisciplinary Primary Care
MD: Medical Doctor
PCN: Primary Care Network
