# Supplementary material for: Funding and remuneration of interdisciplinary primary care teams in Canada: a conceptual framework and application
Source: BMC Health Serv Res. 2017 May 15;17:351. doi: 10.1186/s12913-017-2290-4 (PMC5433058; doi:10.1186/s12913-017-2290-4)
Supplement: Supplementary file 1 — COREQ. Consolidated Checklist for the Reporting of Qualitative Research—Checklist. Description of data: n/a. (DOCX 16 kb) [file 12913_2017_2290_MOESM1_ESM.docx]

**COREQ – Consolidated criteria for reporting qualitative research - Checklist**

Tong A. Sainsbury P. Craig J. Consolidated criteria for reporting qualitative research (COREQ): a 32-item checklist for interviews and focus groups. International Journal for Quality in Health Care. 19(6):349-357

| **Item** | **Guide questions/ description** | **Response** |
| --- | --- | --- |
| ***Domain 1: Research team and reflexivity*** | | |
| *Personal Characteristics* | | |
| 1. Interviewer/ facilitator | Which author(s) conducted interviews or focus groups? | DW (and research assistants= RAs) |
| 2. Credentials | What were the researchers credentials? E.g. PhD, MD | DW hold a PhD, RAs had a undergraduate degree, one a graduate degree |
| 3. Occupation | What was their occupation at the time of study? | DW is an Associate Professor, RAs were graduate students |
| 4. Gender | Was the researcher male or female? | DW is female, one RA is male |
| 5. Experience and training | What experience or training did the researcher have? | DW is an experienced interviewer in professional settings, RAs had graduate level training, and were additionally trained by DW |
| *Relationship with Participants* | | |
| 6. Relationship established | Was a relationship established prior to study commencement? | Some respondents were previously professionally associated with AL, AK, MK, JE or IB. |
| 7. Participant knowledge of the interviewer | What did the participants know about the researcher? E.g. personal goals, reasons for doing the research | Participants were informed about the reasons for the research via the invitation to participate, and the consent form. In addition, the study program has a public website. |
| 8. Interviewer characteristics | What characteristics were reported about the interviewer/facilitator? Bias, assumptions, reasons and interests in the research topic? | Researcher profiles are available on the study website, where professional paradigms are described (e.g. health economist, epidemiologist, decision maker etc.) |
| ***Domain 2: Study design*** | | |
| *Theoretical framework* | | |
| 9. Methodological orientation and theory | What methodological orientation was stated to underpin the study? E.g. grounded theory, discourse analysis, ethnography, phenomenology, content analysis | The study framework was a policy analysis. |
| *Participant Selection* | | |
| 10.Sampling | How were participants selected? E.g. purposive, convenience, consecutive, snowball | The sample was purposive in that we approached participants in the positions of manager or director of IDPC teams. |
| 11. Method of approach | How were participants approached? e.g. face-to-face, telephone, email, mail | Participants were approached via email or telephone. Participants in Alberta were approached via email on a “cold-call” basis, participants in Manitoba and Nova Scotia were approached via email, but knew some of the authors professionally (IB and AL in Nova Scotia, JE and AK in Manitoba) |
| 12. Sample size | How many participants were in the study? | 19 managers/ directors were interviewed. 14 individuals participated in the roundtable, of which 5 were also interview respondents, 4 were also study co-authors, and 5 were new to the study (MK, JE). |
| 13. Non-participation | Now many participants refused to participate or dropped out? Reasons? | One in Nova Scotia, three in Manitoba were unable to commit the time to the study. In Alberta, 42 primary care networks were invited via email, and |
| *Setting* | | |
| 14. Setting of data collection | Where was the data collected? E.g. home, clinic, workplace | Interview data were collected via telephone, respondents were not asked about their physical location. Roundtable data were collected at during the Roundtable event in a board room at Dalhousie University in Halifax, Nova Scotia. |
| 15. Presence of non-participants | Was anyone else present besides the participants and researchers? | During telephone interviews, RAs were accompanied by DW for the first three interviews, then were unaccompanied. Respondents were not asked about the presence of others. During the roundtable, two RAs and one project administrator were present to assist with logistics. |
| 16.Description of sample | What are the important characteristics in the sample? E.g. demographic data, date | Participants were selected, if their professional role was to oversee IDPC team(s) either as managers, or directors. Participants for the roundtable also included policy decision makers. |
| *Data collection* | | |
| 17. Interview guide | Were questions, prompts, guides provided by the authors? Was it pilot tested? | Initial interview questions were developed by DW, AK, AL and discussed by the study team, and validated by co-authors who are policy decision makers in primary care (MK, JE, IB). The questions were also reviewed by appropriate ethics review boards. |
| 18. Repeat interviews | Were repeat interviews carried out? If yes, how many? | On six occasions, follow-up contact was established because initial responses were incomplete or not clear. |
| 19. Audio/ visual recording | Did the research use audio or visual recording to collect data? | All interviews and the roundtable were audio recorded and transcribed. |
| 20. Field notes | Were fields notes made during and/or after the interview or focus group? | RAs took notes during the Roundtable, primarily to assist with the identification of speakers for purposes of transcription. |
| 21. Duration | What was the duration of the interviews or focus groups? | Each interview lasted approximately one hour. The Roundtable was a two day event, for which the schedule is provided as an appendix to the manuscript. |
| 22. Data saturation | Was data saturation discussed? | Interviews in Alberta revealed that the funding/ remuneration approach was uniform across the Province, and the challenges experienced were similar, though varied by size and location of the PCN network. Interviews in Manitoba and Nova scotia revealed a diversity of financing approaches, but relatively similar challenges. It is the conclusion that additional interviews in the three selected provinces would not bring to light additional themes. |
| 23. Transcripts returned | Were transcripts returned to participants for comment and/or correction? | Interview transcripts were verified in case of lack of clarity (addressed under 18. Repeat interviews). Several interviewees provided written response to some of the questions. Transcripts were not provided to respondents in their final versions, and no such promise was made. |
| ***Domain 3: Analysis and findings*** | | |
| *Data analysis* | | |
| 24. Number of data coders | How many data coders coded the data? | DW was responsible for coding, with assistance from RAs. DW verified themes with all study authors. |
| 25. Description of coding tree | Did authors provide a description of the coding tree? | The framework analysis approach was taken and the coding framework (level 1 code) is provided in the Appendix. |
| 26. Deviation of themes | Were themes identified in advance or derived from the data | Interview themes were identified in advance. Evaluative components of the interviews were thematically coded ex post, as were transcriptions from the roundtable. |
| 27. Software | What software, if applicable, was used to manage the data? | Only Word and Excel were used. |
| 28. Participant checking | Did participants provide feedback on the findings? | Roundtable participants were provided with the report version of this study (available online) and were given 30 days to provide feedback. |
| *Reporting* |  |  |
| 29. Quotations presented | Were participant quotations presented to illustrate themes/ findings? Was each quotation identified? E.g. participant number | Quotations are provided but are not attributed to particular respondents / type of respondents. |
| 30. Data and findings consistent | Was there consistency between the data presented and the findings? | Yes. |
| 31. Clarity of major themes | Were major themes clearly presented in the findings? | Major themes are presented and captured in the form of a conceptual framework. The framework is applied to the analysis of the particular issue of collaboration. Four major implementation issues are discussed. |
| 32. Clarity of minor themes | Is there a description of diverse cases or discussion of minor themes? | There were no deviating cases/ and in addition, respondents were informed that only aggregate results would be presented. Minor themes were not discussed to keep focus. |
