# Supplementary material for: Funding and remuneration of interdisciplinary primary care teams in Canada: a conceptual framework and application
Source: BMC Health Serv Res. 2017 May 15;17:351. doi: 10.1186/s12913-017-2290-4 (PMC5433058; doi:10.1186/s12913-017-2290-4)
Supplement: Supplementary file 2 — Qualitative Interview Questions. Description of data: Questions used during qualitative interviews. (DOCX 19 kb) [file 12913_2017_2290_MOESM2_ESM.docx]

**APPENDIX 1 - Qualitative Interview Questions**

**In person interview (for the team/network manager)**

In recent years a large body of literature has arisen supporting the development of interdisciplinary health teams, as they facilitate improved patient outcomes through disease prevention and chronic disease management. Of note, there remain significant gaps in the literature with respect to optimal team compensation and management structure. The purpose of this research is to describe current managerial structures and compensation models for primary health care teams in three Canadian provinces.

Written informed consent must be obtained prior to completing this interview (see Informed Consent Form).

**PART I – Composition and organization of the team/network**

In this section, questions ask about the nature of your interdisciplinary team/network. Teams or networks vary from region to region in the ways that they are organized. Our goal is to clearly understand the composition and organization of your team/network.

**Question 1**

Describe your definition of an interdisciplinary or multidisciplinary team or network. How do we know, who is a member of your team/network and who is not?

**Question 2**

Please describe the strategic goals of your team/network. If none are explicitly stated, please describe the implicit goals.

**Question 3**

Using the matrix provided at the end of this questionnaire, please identify the member of your team/network by the function that they serve.

**Question 4**

How many patients are attached to/ served by your team/network? How is this number determined? Is there a formal or informal patient list (panel)? Is it at capacity?

**Question 5**

Is there anything else that you can tell us about the organizational structure of your team/network? How has it changed through history? Why were these changes made?

**PART II – Governance structure of the team/network**

In this section, questions ask about the governance structure of your team/network. Our goal is to understand what the decision-making processes are, and what kind of a hierarchy exists within your team/network.

**Question 6**

Please describe who is formally (and/or informally) in charge of decision-making with respect to team structure? E.g. who decides that the team/network should expand? Who makes the hiring decisions?

**Question 7**

Please describe who is formally (and/or informally) in charge of strategic planning for the team/network? E.g. who decides if the team/network should focus on particular populations, or on particular disease areas?

**Question 8**

Currently, if any, what are the challenges associated with the governance and management of the team/network? What seems to be working well?

**Question 9**

Is there anything else that you can tell us about the governance structure of your team/network? How has it changed through history? Why were these changes made?

**PART III – Funding arrangements and compensation**

In this section, questions ask about the funding of your team/network. We want to understand how the team/network as a whole is funded, and also how funds are distributed between individual team/network members.

**Question 10**

Please describe how the team/network as a whole is funded?

**Question 11**

Please describe how individual members within the team/network are compensated? Using the matrix provided at the end of this questionnaire, indicate the method of compensation for each type of team/network member.

**Question 12**

Please describe how the decisions were made around compensation methods? E.g. Who decided to pay salaries to specific team/network members, and why?

**Question 13**

Currently, if any, what are the challenges associated with the funding arrangement of the team/network and compensation of individual members? What seems to be working well?

**Question 14**

Is there anything else that you can tell us about the funding arrangements and compensation methods in your team/network? How has it changed through history? Why were these changes made?

**Question 15**

Given your observations of the daily operation of the team/network, please assess whether the current funding arrangements and compensation methods support the goals of the team/network. Please elaborate on the reasons for your assessment.

***Table used to capture the team composition as described by individual respondents.***

| **Province: ________________________ City:_________________________________**  **Clinic Name: _________________________________________________________________** | | | |
| --- | --- | --- | --- |
| **Position** | **Number of Positions** | **Compensation Method*** | **Funding source and contract** |
| Physicians |  |  |  |
| Physician Leads |  |  |  |
| Registered Nurses |  |  |  |
| Licensed Practical Nurses |  |  |  |
| Nurse Practitioners |  |  |  |
| Pharmacists |  |  |  |
| Registered Dieticians |  |  |  |
| Social Workers |  |  |  |
| Medical Office Assistants |  |  |  |
| Psychologist |  |  |  |
| Counselors |  |  |  |
| Midwives |  |  |  |
| Occupational Therapist |  |  |  |
| **Administration** |  |  |  |
| Executive Directors |  |  |  |
| Administrative Assts. |  |  |  |
| Billing Personnel |  |  |  |
| Referral Coordinators |  |  |  |
| **Positions Not Listed Above** |  |  |  |
|  |  |  |  |
|  |  |  |  |
|  |  |  |  |
|  |  |  |  |
