# Supplementary material for: Funding and remuneration of interdisciplinary primary care teams in Canada: a conceptual framework and application
Source: BMC Health Serv Res. 2017 May 15;17:351. doi: 10.1186/s12913-017-2290-4 (PMC5433058; doi:10.1186/s12913-017-2290-4)
Supplement: Supplementary file 3 — Qualitative Analysis Tool. Description of data: The framework used to assist with analysis of qualitative interviews. (DOCX 17 kb) [file 12913_2017_2290_MOESM3_ESM.docx]

**Appendix 2 - Qualitative Analysis Tools**

***Matrix used to organize qualitative interview data***

| **RESPONDENT** | Anonymized (name of respondent not seen) |
| --- | --- |
| **NAME AND LOCATION** | Name of the clinic and specific location (province and region). |
| **ORGANIZATION** | |
| Interdisciplinarity | Their definition, if provided |
| Logistics | Co-located or not, shared resources etc. |
| History | Planning, design, implementation, who was in charge etc. |
| Goals | General goals (are 3C&Q mentioned)   - Explicit or implicit - Who came up with the goal statement   Specific disease areas  Specific populations |
| **COMPOSITION** | |
| Team membership | How many professionals of each type  Team membership (formal, virtual) |
| Patient membership | How many patients  Formal panel/ roster |
| **GOVERNANCE STRUCTURE** | |
| Decision making and conflict resolution | Organization/ composition  Goals, focus  Patient care, day to day decisions  Explicated or implicit processes |
| Respondents’ assessment | Summary of the advantages and disadvantages, merits and demerits of the governance structure discussed in the interview. |
| **FUNDING ARRANGEMENTS AND COMPENSATION** | |
| Funding of team/network | Source of funding  Funding formula  Who made those decisions (and why) |
| Remuneration of individual providers= | Source of funding  Funding formula  Who made those decisions (and why) |
| Respondents assessment | Summary of the advantages and disadvantages, merits and demerits of the funding arrangements and compensation discussed in the interview. |
| **COMMENTS FOR ANALYSIS** | |
| Additional comments made by respondents regarding any of the team organization or financial arrangements discussed during the interview.  Additional comments made by respondents about IDPC teams that were not directly asked in the interview. Emergent issues. | |
