# Supplementary material for: Funding and remuneration of interdisciplinary primary care teams in Canada: a conceptual framework and application
Source: BMC Health Serv Res. 2017 May 15;17:351. doi: 10.1186/s12913-017-2290-4 (PMC5433058; doi:10.1186/s12913-017-2290-4)
Supplement: Supplementary file 4 — Roundtable Agenda. Description of data: Content of discussion during the 2 day research roundtable data collection component. (DOCX 17 kb) [file 12913_2017_2290_MOESM4_ESM.docx]

**Appendix 3 – Roundtable Agenda**

| **Monday October 27^th^, 2014** | | |
| --- | --- | --- |
| ***TIME*** | ***EVENT*** | ***DESCRIPTION*** |
| 8:30 – 9:00 | Breakfast |  |
| 9:00 – 9:15 | Opening | Introduction to the project  Objectives of the roundtable |
| 9:15 – 10:00 | Introductions | Each roundtable participant speaks about their expertise, skills, and interest in this research project for a few minutes. |
| 10:00 – 11:00 | Interim Report | Presentation of the current version of the report, which is based on the results of interviews and document reviews. Discussion to follow. |
| 11:00 – 12:30 | Case Studies 1 | Knowledge users discuss the landscape of interdisciplinary PHC in their Provinces. |
| 12:30 – 13:30 | Lunch |  |
| 13:30 – 15:00 | Case studies 2 | Roundtable participants discuss the landscape of interdisciplinary PHC teams from their perspective in their Provinces. (All participants) |
| 15:00 – 15:15 | Break |  |
| 15:15 – 15:45 | Break-out Discussion | Break-out groups by province will discuss issues specific to their context. Each session facilitated by a member of the research team. |
| 15:45 – 15:45 | General Discussion | Similarities and differences between provinces, including plans, status-quo, and perceived challenges (focus on: funding/ remuneration/ governance). |
| **Tuesday, October 28^th^, 2014** | | |
| ***TIME*** | ***EVENT*** | ***DESCRIPTION*** |
| 8:30 – 9:00 | Breakfast |  |
| 9:00 – 9:30 | Re-cap | Summary of first day. |
| 9:35 – 11:30 | Implementation issues | Panel presentation of implementation issues from operational, clinical and policy perspectives. Discussion to follow. (Panelists names confidential) |
| 11:35 – 12:30 | Measuring Success | Discussion of what success would look like in primary care. What kinds of measures should an assessment of effectiveness address? (All participants) |
| 12:30 – 13:30 | Lunch | Several attendees depart. |
| 13:30 – 15:00 | Discussion | Informal discussion of the current research results and research plans for future projects. |
